# Particulate metal exposures induce plasma metabolome changes in a commuter panel study

**DOI:** 10.1371/journal.pone.0203468

**Published:** 2018-09-19

**Authors:** Chandresh Nanji Ladva, Rachel Golan, Donghai Liang, Roby Greenwald, Douglas I. Walker, Karan Uppal, Amit U. Raysoni, ViLinh Tran, Tianwei Yu, W. Dana Flanders, Gary W. Miller, Dean P. Jones, Jeremy A. Sarnat

**Affiliations:** 1 Department of Epidemiology, Rollins School of Public Health, Emory University, Atlanta, GA, United States of America; 2 Department of Public Health, Ben-Gurion University of the Negev, Beer Sheva, Israel; 3 Department of Environmental Health, Rollins School of Public Health, Emory University, Atlanta, GA, United States of America; 4 Department of Environmental Health, Georgia State University, Atlanta, GA, United States of America; 5 Clinical Biomarkers Laboratory, Division of Pulmonary, Allergy, and Critical Care Medicine, School of Medicine, Emory University, Atlanta, GA, United States of America; 6 Department of Biostatistics and Bioinformatics, Rollins School of Public Health, Emory University, Atlanta, GA, United States of America; Boston University, UNITED STATES

## Abstract

**Introduction:**

Advances in liquid chromatography-mass spectrometry (LC-MS) have enabled high-resolution metabolomics (HRM) to emerge as a sensitive tool for measuring environmental exposures and corresponding biological response. Using measurements collected as part of a large, panel-based study of car commuters, the current analysis examines in-vehicle air pollution concentrations, targeted inflammatory biomarker levels, and metabolomic profiles to trace potential metabolic perturbations associated with on-road traffic exposures.

**Methods:**

A 60-person panel of adults participated in a crossover study, where each participant conducted a highway commute and randomized to either a side-street commute or clinic exposure session. In addition to in-vehicle exposure characterizations, participants contributed pre- and post-exposure dried blood spots for 2-hr changes in targeted proinflammatory and vascular injury biomarkers and 10-hr changes in the plasma metabolome. Samples were analyzed on a Thermo QExactive MS system in positive and negative electrospray ionization (ESI) mode. Data were processed and analyzed in R using apLCMS, xMSanalyzer, and limma. Features associated with environmental exposures or biological endpoints were identified with a linear mixed effects model and annotated through human metabolic pathway analysis in mummichog.

**Results:**

HRM detected 10-hr perturbations in 110 features associated with in-vehicle, particulate metal exposures (Al, Pb, and Fe) which reflect changes in arachidonic acid, leukotriene, and tryptophan metabolism. Two-hour changes in proinflammatory biomarkers hs-CRP, IL-6, IL-8, and IL-1β were also associated with 10-hr changes in the plasma metabolome, suggesting diverse amino acid, leukotriene, and antioxidant metabolism effects. A putatively identified metabolite, 20-OH-LTB4, decreased after in-vehicle exposure to particulate metals, suggesting a subclinical immune response.

**Conclusions:**

Acute exposures to traffic-related air pollutants are associated with broad inflammatory response, including several traditional markers of inflammation.

## Introduction

Globally, source apportionment studies attribute 25% of urban ambient particulate matter less than 2.5 microns (PM_2.5_) to traffic sources [1]. Nearly ubiquitous, traffic-related pollution (TRP) has been linked to numerous adverse health effects [2]. Despite this, the specific constituents of TRP responsible for these effects and how they contribute to corresponding biological responses are still not well understood [3]. Uncertainty regarding the etiology of TRP-related toxicity is, in part, due to the complexity of exposures to this pollutant source, as well as the numerous biological pathways that may mediate response [4–7]. It is possible that more sensitive measures of both exposure and response may help identify critical components of TRP and their complementary pathways impacting human health.

Environmental metabolomics has emerged as an approach for sensitively quantitating thousands of chemical signals in a biological sample, providing broad spectrum measurements of human metabolism that may be indicative of environmental chemicals [8]. Metabolomic perturbations have been associated with occupational air pollution exposures [9, 10]. Walker, et al., employed high-resolution metabolomics (HRM), using sensitive liquid chromatography-mass spectrometry (LC-MS), coupled with advanced bioinformatics methods, as a platform linking exposure to internal doses and biological responses. Occupational exposures to trichloroethylene and likely metabolic products were measurable in blood and correlated with expected detoxification pathways [10]. Recently, ambient air pollution exposures have also been found to associate with metabolic perturbations in humans using untargeted approaches [11].

Panel studies of commuting populations provide an exceptional platform to observe potential acute effects of traffic pollution in humans using realistic exposures. This design can harbor the strength of high contrasting exposures [12], while limiting risk to participants to what may be experienced in real-world contexts [13]. We have previously employed panel-based designs, with quasi-experimental exposures, in targeted biomarker analyses following exposures to TRP [13–15]. By introducing repeated biological sampling, researchers can disentangle short term changes in key biological endpoints, such as inflammation and lung function [16] and potentially reveal new insights on TRP toxicity in humans.

The present analysis leverages extensive measurement collected within a scripted, longitudinal panel of car commuters and novel HRM profiling to investigate potential perturbations of the plasma metabolome following exposures to TRP. The Atlanta Commuters Exposure (ACE) panel study included a targeted examination of oxidative stress and inflammation for a suite of cardiovascular and respiratory outcomes associated with on-road traffic exposures during morning rush hour car commutes in Atlanta, GA [17, 18]. In-vehicle exposures to PM_2.5_, its components, and noise were measured for 60 participants along with repeated biological sampling of numerous heart, lung, and inflammation-related endpoints. Here, we present results examining associations between: 1) specific TRPs with known markers of inflammation and lung function, including forced expiratory volume and high sensitivity C-reactive protein; 2) TRP with perturbations of the metabolome measured twice over a 10h duration; and 3) targeted markers of inflammation and lung function with perturbations of the metabolome. The rationale for the approach arose from the breadth and depth of information available to the researchers from the ACE population and a need in the field to compare hypothesis-driven, targeted measures of response to hypothesis-free, untargeted measures of response. The strength of this analysis is centered around the presence of both known, targeted markers of biological response, with unknown, targeted metabolic features critical for establishing discovery-phase science, as well as a highly-speciated personal exposure assessment for each participant, often lacking in human observational studies.

## Materials and methods

### Study design

The ACE study was a longitudinal panel of 60 participants conducted in Atlanta, GA from 2011 to 2013. The design, participant characteristics, and exclusion criteria have been previously discussed [17–19]. This analysis focuses on a subset of 49 participants within the complete panel for which plasma samples were available. In brief, the study participants were measured for inflammatory and cardiorespiratory responses before and after conducting two of three distinct exposure scenarios. The three, 2-hr exposure scenarios were highway, side street, and indoor clinic environments during morning rush hours (approximately 7 to 9 AM). Each was scheduled a week apart and one was necessarily a highway exposure. The study protocol and materials were approved by the Institutional Review Board of the Rollins School of Public Health of Emory University (IRB Study Number 47904). All participants provided written informed consent.

### Exposure assessment

In-vehicle and clinic pollutant sampling was conducted during the exposure period as described previously [20] with characterizations previously published [17]. We focused on particulate matter and specific particulate components with the potential to elicit oxidative stress in humans. We measured PM_2.5_ mass, black carbon (BC), particle-bound polycyclic aromatic hydrocarbons (pb-PAHs), particle number concentration (PNC), and noise (dB) continuously using instrumentation housed in a sampling apparatus located in the passenger seat or clinic room during sampling periods [21]. These continuous measures, captured in 1-second intervals, were time-averaged to 1-minute concentrations for each TRP. We also extracted particulate mass from either quartz or Teflon substrates to characterize 2-hr integrated elemental metal (Al, Pb, and Fe), total organic carbon (OC), and water-soluble organic carbon (WSOC) concentrations. Elemental concentrations (pg/mL) and carbon fraction concentrations (μg/m^3^) were measured using ICP-MS and TD-GC-MS, respectively. This subset of pollutants was chosen *a priori* based upon previously demonstrated associations with traffic or mobile source emissions [18, 21].

### Biological sampling

Sampling and targeted biomarker analysis are outlined in detail elsewhere [17]. Briefly, a panel of inflammatory and oxidative stress markers was collected at multiple time points before and after each commute. Respiratory markers exhaled nitric oxide (eNO), forced expiratory volume (FEV_1_) and blood-based markers were collected concurrently at both pre-exposure (7AM) and post-exposure (9AM). Dried blood spots were analyzed for high-sensitive C-reactive protein (hs-CRP), interleukin-6 (IL-6), interleukin-8 (IL-8), interleukin-1β (IL-1β), tumor necrosis factor α (TNF-α), soluble intercellular adhesion molecule (sICAM) and soluble vascular cell adhesion molecule (sVCAM) using multiplexed inflammatory and vascular injury panels (Luminex). eNO was measured using a NIOX Mino (Circassia) and FEV_1_ was measured using a handheld spirometer. The present analysis focuses on the differences between post- (9AM) and pre-exposure (7AM) measurements of these respiratory and inflammatory markers (ΔBiomarkers). Previously, our group showed that lung function markers changed immediately post-exposure from baseline while temporal patterns of blood-based markers were not statistically significant at any time point [17]. In choosing the 7AM and 9AM measures, we believed to capture a temporal window that most closely matched the exposure measures (ΔBiomarkers and Exposure are data combined in S1 Datasets).

Whole, venous blood was collected pre-commute (7AM) and post-commute (6PM). Blood was collected from the arm, usually the cubital vein, into purple topped containers (containing ethylenediamineacetic acid). Samples were spun, and plasma supernatants were aliquoted immediately after collection. All plasma was stored at -80°C.

### High-resolution metabolomics

Metabolomics was completed using established methods [8, 22]. Plasma samples were diluted two-fold with acetonitrile and analyzed in triplicate using a dual-chromatography, high-resolution mass spectrometry system (Dionex Ultimate 3000; ThermoScientific QExactive). Analyte separation was accomplished using reverse-phase C_18_ liquid chromatography (Targa C_18_ 2.1mm x 100mm x 2.6μm, Higgins Analytical) with mass spectral detection completed in positive and negative mode electrospray ionization at 70,000 (FHWM) resolution over a mass-to-charge ratio (m/z) range of 85 to 1250. For quality control, all sample batches included two replicates of a pooled reference material. NIST SRM 1950 was also included at the beginning and end of all study samples. Concentrations of select metabolites were determined by reference standardization using the positive mode data only [23].

Raw data files were processed in R for feature extraction and quality control using the hybrid mode of adaptive processing of liquid chromatography mass spectrometry (‘apLCMS’) [24] with modifications by ‘xMSanalyzer’ [25] to generate the final feature tables used for analysis (Final tables within S1 Datasets). To improve detection of low abundance metabolites and environmental chemicals, a reference data base of known metabolites was provided to apLCMS which comprised of blood metabolites from the Human Metabolome Database v. 3.6 and a list of 1,209 compounds from the Environmental Protection Agency’s Mobile Source Air Toxics Inventory. Detected features were defined as a unique ion identified by its m/z, retention time (RT), and intensity. This algorithm was run separately for each of the positive and negative mode raw spectrograms with respective lists of ion adducts optimized for the metabolomics platform. Only those features that were reproduced over two, distinct iterations of apLCMS, exhibited median coefficients of variation (CoV) across technical triplicates less than 30%, and were detected in at least 10% of the biological samples were included in the statistical analysis. Values below the analytical limit of detection (LOD) for each feature were imputed with LOD/2.

### Statistical analysis

Microenvironmental concentrations were first compared with corresponding changes in targeted biomarker levels with subject as a random effect using mixed effect models to explore direct association between exposure and targeted biological responses [17]. Metabolomics data were analyzed in two ways, focusing first on environmental (Exposure_ij_) associations, and then on targeted biomarker (ΔBiomarker_ij_) associations. The approach comprised feature selection by metabolome wide association (MWAS) and human metabolic pathway enrichment of significant features performed on each ion mode separately [10].

Statistical analyses were conducted using the R Statistical Platform (v. 3.3.1) packages ‘xMSanalyzer,’ ‘apLCMS,’ and ‘limma’ [25–27]. Associations between TRP and the targeted biomarkers were conducted using linear mixed effect regression models described, in detail, previously [17]. HRM data were first log2-transformed before being analyzed using the R statistical package ‘limma.’ ‘Limma’ is designed to run mixed effects models on high dimensional data and moderate t-statistics using an empirical Bayes method for shrinking standard errors [26]. The package was created for RNA sequencing analyses; however, the data have structural similarities. For example, RNA sequencing data is a collection of color intensities at specific probes and spectral data is a collection of signal intensities at specific mass to charge ratios.

The chosen model specifications examine the associations between the fluxes of metabolomic profiles at 10 hours apart with either exposure during the ‘commute’ period or the flux of targeted biomarkers around the commute period. All models included categorical co-variates to control for the potential effects of asthma status, age, sex, body mass index (BMI), and race without explicit control of the exposure setting. We first modeled associations between pre- to post-exposure changes in metabolite intensities (‘ΔFeature_ij_’) and corresponding pollutant exposure metrics (Exposure_ij_) (Model 1). Directly measured pollutants, PM_2.5_, OC, BC, WSOC, pb-PAHs, PNC, noise, Al, Fe, and Pb, were examined, in turn, as continuous variables.

$${\Delta Feature}_{ij}=\mu +\theta_{i}+\beta_{1}{Exposure}_{ij}+\beta_{2}{Asthma}_{i}+\beta_{3}{Age}_{i}+\beta_{4}{Sex}_{i}+\beta_{5}{BMI}_{i}+\beta_{6}{Race}_{i}+\varepsilon_{ij},$$ (Model 1)

where *j* indexes commute day, *i* indexes subject, $\theta_{i}∼N(0,\pi ),\varepsilon_{ij}∼N(0,\sigma^{2}),and\;\pi ={\left(\frac{\sum_{1}^{k}\tau^{2}}{k}\right)}_{15\%}$ ‒the trimmed mean of intra-individual variability.

The second model examined associations between ΔFeature_ij_ and corresponding pre- and post-exposure changes in eNO, FEV_1_, hs-CRP, TNF-α, IL-1β, IL-6, IL-8, sICAM, and sVCAM (ΔBiomarker) (Model 2).

$${\Delta Feature}_{ij}=\mu +\theta_{i}+\beta_{1}{\Delta Biomarker}_{ij}+\beta_{2}{Asthma}_{i}+\beta_{3}{Age}_{i}+\beta_{4}{Sex}_{i}+\beta_{5}{BMI}_{i}+\beta_{6}{Race}_{i}+\varepsilon_{ij},$$ (Model 2)

where indexing of variables and errors are as described for Model 1.

Each set of results—defined by the combination of primary predictor and ion mode—contained *p*-values and moderated t-statistics for all ΔFeature_ij_. The *p*-values were adjusted for multiple comparisons using the Benjamini-Hotchberg false discovery rate (FDR_B-H_) at *FDR*_*B-H*_ < 0.05 [28]. We used Manhattan plots of the -log_10_(*p*) over feature retention time with the average direction of change between post- and pre-exposure metabolite intensities as a means of visualizing significant associations from the mixed effects models.

### Human metabolic pathway enrichment and putative feature identification

We conducted pathway analysis using mummichog (v. 1.0.7) and ran separate analyses for each set of features from the linear mixed models using both positive and negative mode features [29]. Mummichog takes provided features and tests modules of in predefined networks and the biological pathways to statistically test presence of significant features on networks of human metabolism, permitting increased confidence in feature identification. Lists of significant features from MWAS were identified with an FDR_B-H_ < 0.05 and further restricted for peak quality by extracted ion chromatograph (EIC) filtering. The human reference pathway for mapping came from MetaFishNet [29], a compilation of KEGG, Edinburgh Human Metabolism Network, UCSD BiGG, and BioCyC network metabolism models. Matches to the pathways were made within 10 ppm to measured feature m/z ratios. Pathways were considered strong candidates if at least 3 nodes from the experimental data overlapped with pathway nodes and the mummichog permutation-based enrichment score, s, was less than 0.10. Human metabolic pathway enrichment results were compared within and across exposure and biomarker set lists for overlapping pathway enrichments.

## Results

Forty-nine of 60 participants (82%) in the ACE study provided venous blood over 73 different sampling days. Participants not contributing blood samples were those enrolled in the study prior to the approval of the venous blood collection protocol. Sixty-nine of those 73 sampling days (95%) were complete samplings, meaning blood was collected both pre- and post-exposure for the individual on a given sampling day. This subpopulation formed the final analytical subset, for whom matching respiratory, inflammatory, and oxidative stress markers were also available. Population characteristics and exposures are summarized in Table 1 and Table 2, respectively. Broadly and as reported previously [13, 17], exposures to many of the TRP exposures were elevated in commutes relative to ambient concentrations. Some particulate pollutants and noise differed between highway and non-highway commutes, but not metals or WSOC (Table 2).

**Table 1:** Participant heath characteristics.

Participant Characteristics	
N	49
Age in years	26 (5)
Female	47%
Race	
White	64%
Asian	20%
Other	16%
Health Status	
BMI (kg·m^2^)	23.15 (3.53)
Asthma Diagnosed	53%

Values are mean (SD), unless noted otherwise.

**Table 2:** Mean in-vehicle exposures by commute type.

Exposure Characteristics	
Commute Type (N)	
Highway	36
Non-Highway	37
PM_2.5_ (μg·m^-3^) ^a^	
Highway	17.14 (6.18)
Non-Highway	11.18 (8.58)
BC (μg·m^-3^) ^a^	
Highway	5.33 (2.23)
Non-Highway	1.58 (1.43)
OC (μg·m^-3^) ^a^	
Highway	7.66 (1.98)
Non-Highway	6.07 (1.70)
WSOC (μg·m^-3^)	
Highway	8.48 (3.75)
Non-Highway	7.95 (3.45)
PNC (#·m^-3^) ^a^	
Highway	34,808 (12,918)
Non-Highway	10,649 (8,147)
pb-PAH (μg·m^-3^) ^a^	
Highway	113.93 (30.51)
Non-Highway	65.21 (38.94)
Noise (dBA) ^a^	
Highway	68.59 (2.73)
Non-Highway	58.40 (11.23)
Aluminum (Al) (ng·m^-3^)	
Highway	29.36 (28.67)
Non-Highway	24.37 (19.71)
Iron (Fe) (ng·m^-3^)	
Highway	176.33 (171.19)
Non-Highway	121.55 (115.99)
Lead (Pb) (ng·m^-3^)	
Highway	0.45 (0.45)
Non-Highway	0.92 (1.64)

Values are mean (SD), unless noted otherwise.

^a^denotes p < 0.05 for Student’s *t* test

**Fig 1:**
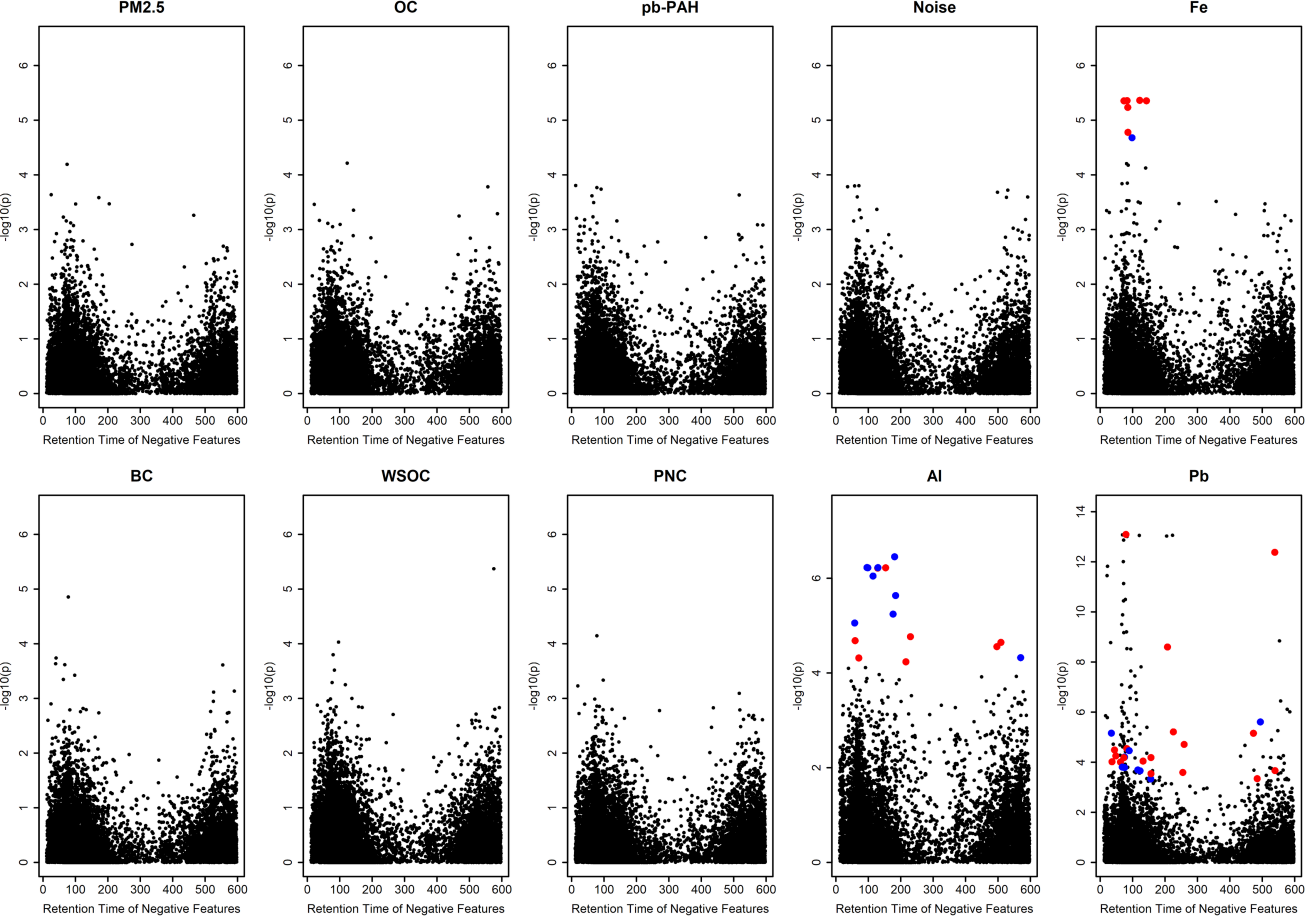
Manhattan plots of associations between changes in negative ionization mode feature intensities with in-vehicle, traffic-related pollutants. Colored points are significant at FDR_B-H_ < 0.05 and indicate average increase (red) or decrease (blue) in feature intensity.

Pre-commute concentrations of select plasma metabolites are provided in S2 Table, confirming reliability of metabolomic profiling because the concentrations of known metabolites were within previously reported levels in a human population [30]. Specific amino acids were above or below reported mean ranges in HMDB. Specifically, the study population comparatively lower in asparagine, citrulline, glutamine, leucine/isoleucine, and tryptophan while glutamate and proline were comparatively higher than HMDB values. Plasma concentrations of eight amino acids and carnitine differed between asthmatics and non-asthmatics. The MWAS of exposure included both positive (S4 and S5 Figs) and negative (Fig 1 and S3 Fig) ionization modes. The positive mode captured 7,390 features with a median CoV of 13.5%. The negative mode included 14,341 detected features with a median CoV of 14.5%.

Associations between TRP and ΔBiomarkers were largely consistent with the null (Table 3). Pb, however, was negatively associated with Δhs-CRP (β = -20.1%; p < 0.005) and ΔsICAM (β = -24.2%; p < 0.001) indicating, on average, a 22% reduction in the acute phase protein and intercellular adhesion molecule-1 per 1 ng/m^3^ increase in Pb exposure. We observed smaller reductions in ΔIL-8 with increases in pb-PAH, PNC, and noise (p < 0.05). We did not observe association with ΔIL-6 and any of the exposure measures used. Notably, many IL-6 levels were distributed below analytic detection (n = 32, 46% below limit of detection) (S2 Table).

**Table 3:** Percent change in biomarker per unit increase in exposure.

	Δhs-CRP	ΔTNF-α	ΔIL1β	ΔIL6	ΔIL8	ΔsICAM	ΔsVCAM	ΔeNO	ΔFEV1
BC	0.87	-2.09	-1.72	-2.18	-2.10	-0.08	0.50	-0.32	-0.32
OC	4.97	-1.53	-8.62	-6.35	*-3*.*21*	9.50	9.98	-2.02	-0.47
WSOC	1.88	2.29	-0.57	—	0.05	1.36	2.64	-0.27	0.12
pb-PAH	-0.10	-0.04	-0.16	-0.46	-0.23^a^	-0.03	0.07	-0.06	-0.02
PNC	0.50	-0.08	<0.01	-0. 78	-0.55^a^	0.58	0.61	-0.20	<-0.01^a^
Noise	-0.63	-0.77^a^	-1.07	1.21	-0.80^a^	-0.85	-0.54	-0.14	-0.02
Al	-0.05	-0.13	0.42	0.37	0.07	-0.30	-0.23	0.05	-0.02
Fe	-0.03	-0.04	0.01	-0.09	-0.01	-0.04	-0.02	<-0.01	-0.01
Pb	-20.14^a^	0.84	-7.13	-5.15	0.81	-24.21^a^	-31.09	-0.16	0.07

PNC % change reflect change in concentration in thousands (1,000s) of particles

^a^Indicates p-value significant at α < 0.05;—indicates a model that did not converge

**Fig 2:**
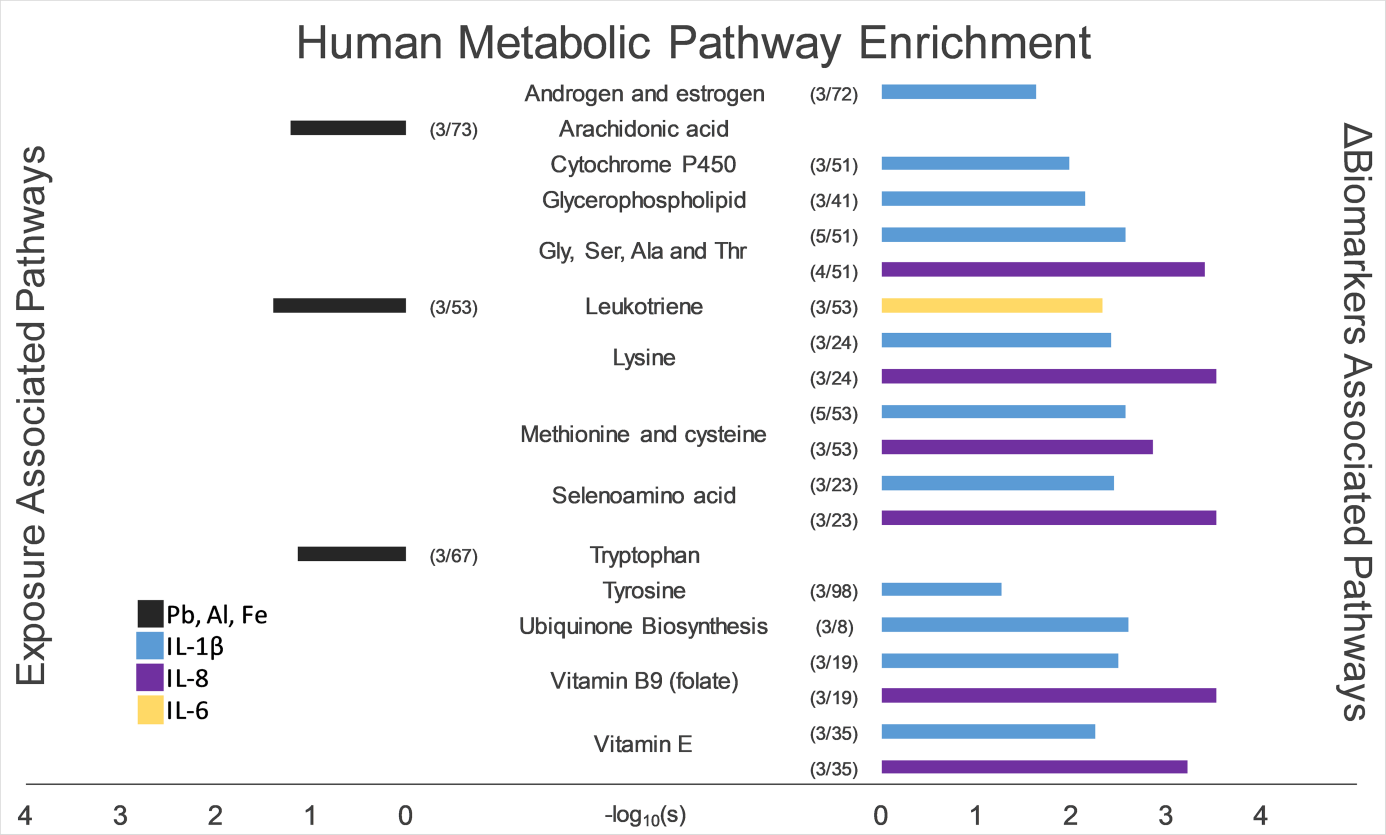
**Pathway enrichment of exposure-based (left) and biomarker-based (right) significant features.** Colored bars indicate the -log_10_(s) of enrichment scores from mummichog, a network-based pathway analysis tool. Numbers in parentheses indicate the ratio of matching features onto a human reference pathway.

Many TRP exposures were not associated with ΔFeature_ij_; however, concentrations of particulate metals Al, Fe, and Pb were associated (FDR_B-H_ < 0.05) (Fig 1; S4 Fig). In the positive mode, 42 total features were associated with either Pb or Fe. MWAS of the negative mode identified 11, 6, and 91 features significantly associated (FDR_B-H_ < 0.05) with Al, Fe, and Pb, respectively (Fig 1). Overall, the average change in concentrations showed a decrease from pre-commute levels for all metal-associated features. As a means of identification of these significant features, we matched the masses of the features with metabolic networks through a pathway analysis (Negative mode: Fig 2). Mummichog was unable to identify putative pathways with any statistical significance for Al-associated and Pb-associated features (p ~ 1.0) from the positive mode nor converge for other pollutant models, likely due to the small number of features identified through the MWAS modeling. However, the negative mode features were more successful at mapping onto reference pathways. Al-associated features enriched for arachidonic acid and leukotriene biosynthesis. Three Al-associated features map onto the leukotriene biosynthesis pathway, which has a membership of 53 nodes (s < 0.04). 20-OH-leukotriene B4 (molecular weight: 352.225 Da) was a top predicted metabolite and, on average, decreased in intensity in the population over time.

Prior to the pathway analysis, correlations of ΔBiomarkers with significant features from the combined set of exposure-associated ΔFeature_ij_ was conducted. We did not observe significant correlations with exposure-associated ΔFeature_ij_ after multiple hypothesis test correction. However, in the ΔBiomarkers MWAS, changes in pro-inflammatory cytokines and acute-phase hs-CRP, but not respiratory or lung oxidative stress markers, were associated with ΔFeature_ij_ (S3 Fig). IL-6, hs-CRP, IL-1β, and IL-8 had 60,47, 47, and 53 significant features across both ionization modes, respectively. There was substantial overlap in features between IL-6 and hs-CRP separately between IL-1β and IL-8. A single feature was shared across all four models. (S2 Fig). Enriched pathways associated with these biomarkers included amino acid metabolism, leukotriene metabolism, and ubiquinone biosynthesis.

**Fig 3:**
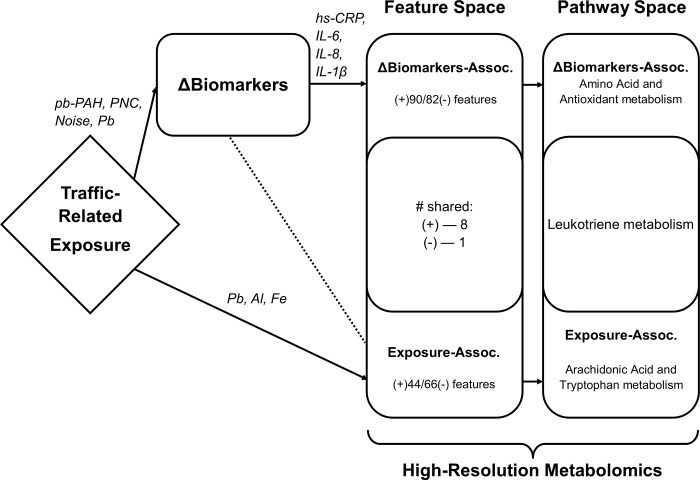
Representation of combined results of MWAS and pathway enrichment of both Exposure and ΔBiomarkers. Significant predictors or enriched pathways (p < 0.05 or s < 0.10) are explicitly named. Particulate metal exposures were the exclusive in-vehicle, traffic related pollutants associated with changes in 110 features of the plasma metabolome. Few significant features overlapped between Exposure-associated and ΔBiomarkers-associated features. Leukotriene metabolism was enriched from Al-associated features and ΔIL-6-associated features.

We summarized the significant results schematically (Fig 3). Overlap between Exposure-associated and ΔBiomarkers-associated MWAS was minimal, showing very few shared features and only one shared, enriched pathway.

## Discussion

Ascertaining TRP components that elicit adverse health response has been challenging. The absence of exposure science tools capable of cost-effectively capturing both environmental exposures and biological responses in humans may be filled through HRM. In the present study, we augmented exposure characterization of an in-vehicle environment and biomonitoring of oxidative stress and inflammation in a small human panel with plasma HRM. A key observation was the association between particulate metal exposures, especially Pb, with 2-hr changes in targeted inflammatory markers (hs-CRP and sICAM) and 10-hr associations with metabolic perturbations in lipid mediators of inflammation and nucleotide driven antioxidation. This is one of the first indications of a directly-measured traffic pollutant associated with changes in human metabolomic profiles. Moreover, we believe these current findings support the utility of HRM as creating multi-pollutant indicators of acute xenobiotic exposure within panel study designs [10, 31].

Collectively, we showed that enriched pathways overlap or are complementary to inflammatory and redox reactions (Fig 3). Three key insights arose from these data: 1) particulate metals or sources containing metals from a morning commute are consistent with an oxidative stress response; 2) functional analysis of significant metabolomic features showed 2-hr changes of inflammatory cytokines and acute-phase protein to be associated with antioxidant pathways; and 3) that, in this human panel, HRM reflected some acute effects of traffic exposure, provided a case-crossover design and a narrow temporal window.

Observed associations across exposures from MWAS suggest covariance between particulate metals in the traffic-related exposures. These metals may serve as tracers for a traffic-related dust source. Potential sources identified from commuter studies by our group include ‘crustal’, ‘non-tailpipe emission’, ‘resuspended road dust’, or ‘brake pad and tire wear’ as descriptors of components of the traffic pollution mixture in vehicle cabins. These similar, complex sources share qualities such as enrichment of Al, Fe, and transition metals [18, 21]. Combining both ACE commuter studies, Krall, et al., reported robust associations of ‘non-tailpipe emissions’—enriched with several metals like Fe—with pulmonary response in the susceptible, asthmatic population. Together, these highlight the elevated presence of metals in a roadway commuting environments and the potential for these pollutants to elicit biological responses through oxidative stress.

Particulate metals have been identified as contributors to cardiovascular disease morbidity [32] and mortality [33]. Transition metals, such as Fe, are redox-active—where the metal ion can serve as both electron acceptor and donor in reactions to generate radical ions. Such metals can promote oxidative stress through redox cycling or quenching antioxidant capacity [34]. These toxic processes have demonstrated inductions by near roadway PM. Pardo, et. al., found both PM extracts from a roadside monitor and a simulated metal solution, including Al, Fe, and Pb, increased IL-6 and TNF-α in murine bronchoalveolar fluid over a 24-hour period with returns to baseline by the 48-hr measurement [35]. In another l mouse model, acute instillation of urban air particles (at 1mg/kg) resulted in 3-hr increases in TNF-α and IL-6 with resolution before a 24-hr measurement [36]. In contrast, ΔTNF-α and ΔIL-6 were not associated with any metal exposures in our commuting population, but Pb was associated with Δhs-CRP and ΔsICAM. For each unit increase in Pb exposure, the percent change in these biomarkers averaged a 22% decrease over a 2-hr period. This decrease is contrary to expectation [37, 38]. One possible explanation may be the potential confluence of circadian patterns of inflammatory markers and the rapid resolution of inflammatory signaling [36] that may result in our observed depression. Our findings indicate that increased short-term metal exposures, specifically Pb, may be responsible for an acute inflammatory response that resolves within a 10-hr window in humans.

The metabolomics analyses suggest that a morning exposure to particulate metals elicit detectable perturbations in leukotriene, arachidonic acid, and tryptophan metabolism over a 10-hr period. Association of tryptophan metabolism with Pb exposures is consistent with the evidence of lead, although redox-inactive, participating in the depletion of antioxidants [34, 39]. While Al is also redox-inactive, it does demonstrate the ability to shift biological systems into oxidative stress [40]. Of Al-associated features, two (m/z: 350.2105 and 350.2102) drive the enrichment of leukotriene and arachidonic acid pathways and suggest an average decrease of these features across the study population after controlling for potential confounders and asthma status over the 10-hr period. These two features match a metabolite of proinflammatory chemoattractant leukotriene B4 (LTB4): 20-OH-LTB4. IL-6-associated features also enriched for leukotriene pathways, providing a concordance with the exposure-based enrichments. Metabolites of LTB4, 20-OH-LTB4 and 12-oxo-10,11-dihydro-20-COOH-LTB4, were putatively identified in mummichog.

**Fig 4:**
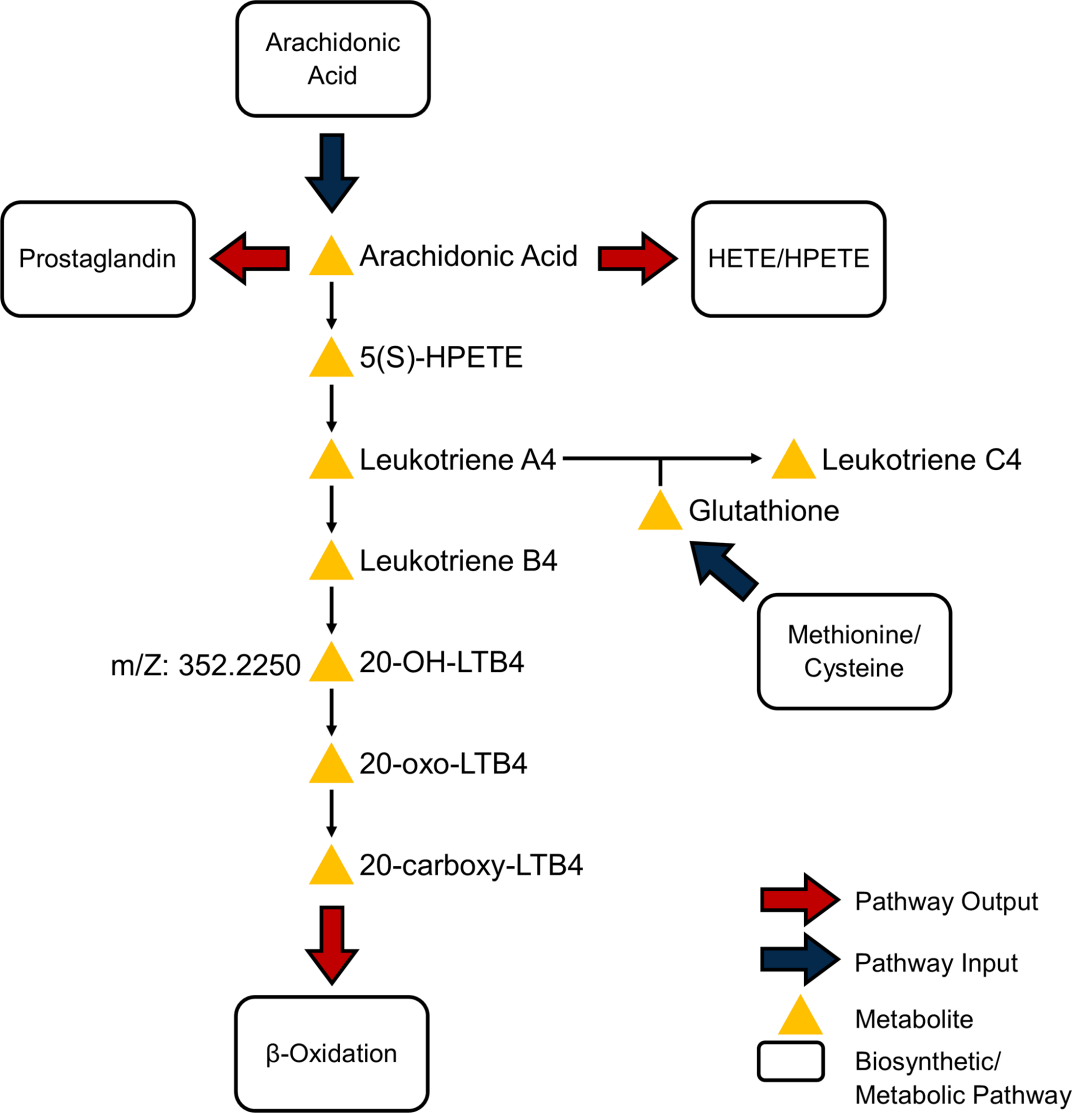
Pathway of leukotriene biosynthesis and catabolism in humans. Leukotriene metabolism was the only pathway to be enriched for in both exposure-based and biomarker-based models using mummichog (overlap ≥ 3 and s ≤ 0.10). The features selected with Al, Pb, and ΔIL-6 models have overlapping matches on this pathway with 20-OH-LTB4. The metabolite putatively detected is a biologically inactive form of leukotriene B4. Arachidonic acid metabolism and glutathione synthesis feed into the pathway to generate the variety of signaling molecules on this pathway. Adapted from MetaCore by Thomson Reuters.

Leukotriene B4 in breath has been associated with the inflammatory response to traffic exposures [41], where long-term traffic exposures, assessed by land use regression models estimating exposure to PM_2.5_, were shown to be associated with increased LTB4 measured in induced sputum (2μg/m^3^ PM_2.5_: ~23% CI_95%_(4%-42%) LTB4). Some attention has grown for leukotrienes in response to TRP exposures. Rabinovitch, et al., (2016) recently reported increases (24% CI_95%_(1.5%,51.5%)) in urinary cysteinyl leukotriene (LTE4) after very short-term exposures to PM_2.5_ ≥ 5μg/m^3^ in asthmatic children [42]. The cysteinyl leukotrienes, LTC4, LTD4, and LTE4, are created from the enzymatic reactions of LTA4 and glutathione (Fig 4). These leukotrienes operate extracellularly, binding to receptors of neighboring cells and promote vascular permeability [43].Putatively identified 20-OH-LTB4 was associated with both Al exposures and ΔIL-6 in our metabolomics population. Δhs-CRP-associated features had substantial overlap with ΔIL-6-associated features (S2 Fig), and enriched for leukotriene metabolism, but not with at least 3 features mapping onto the pathway. Together, these results suggest inactivated leukotrienes in various forms are responsive to TRP exposure.

Outside of the metabolomics analysis, associations between TRP exposure and changes in targeted biomarkers (ΔBiomarkers) were not consistent between the entire ACE population and the subset for which plasma blood draws were available (*results not shown*). In the total ACE population, OC was the only pollutant associated with biomarkers in dried blood spots (Δhs-CRP and ΔsVCAM). This association was not reproduced in the blood draw subset. Other pollutants (pb-PAH, PNC, noise, and lead) were associated with ΔIL-8, ΔsICAM, Δhs-CRP, and ΔTNF-α in the blood draw subset and not in the total ACE population. This discrepancy is indicative of the challenges faced in air pollution studies focusing on hypothesized, targeted biomarkers of effect on inflammatory and oxidative stress pathways [3, 44]. Within our study, plausible reasons exist for the discrepancies between the full data and the subset including1) the presence of false positives due sampling of whole blood after the already study began; 2) technician measurement error in sample handling and processing; or 3) the chance, natural variability of the markers measured. Despite these differences, the addition of HRM with our workflow afforded consistent insights by measuring changes in the human plasma metabolome (ΔFeature) at relevant time scales and capturing markers used in air pollution studies.

There are other caveats and limitations of this analysis that warrant attention and have the potential to inform future air pollution metabolomic study designs. Importantly, metabolic expression is sensitive to inter-personal variability [45]. We controlled, at least in part, for diurnal variation in biomarker levels, participant sex, age, asthma status, obesity, and metro Atlanta residency, although unspecified confounding may be still be present. Our choice to define ΔBiomarkers as the difference in measurements from pre-commute (7AM) to post-commute (9AM) was done to reduce the likelihood of capturing responses due to unmeasured exposures, especially since temporal trends in the cytokine markers were not significant from baseline measures [17]. Sartini, et al., demonstrated in an older male population that IL-6, a key measure of our study, increased almost linearly over a day but without relevant impact on cardiovascular disease risk [46]. In contrast, hs-CRP has been reported to be at most minimally associated with time of day [47, 48]. Repeated measures and contrasting exposures within study participants was a strength where changes in measurements over time can be explored with partitioning of inter- and intra-individual variability [49], and may have provided analytical improvement over cross-sectional analyses for causal inference [9].

The ACE study protocol included self-reported food intake over the study days, but did not explicitly control diet (except for the consumption of nitrate rich foods and leafy greens). Thus, aspects of diet may still contribute confounding. A future analysis may consider including dietary food logs, nutritional assessments, or standards of common foods known to promote pro-inflammatory responses in humans [50].

As a first step to using HRM in air pollution panel studies, our motivation was to find the strongest evidence of an environmental exposure predicting changes in the metabolomes. At the MWAS stage, features were significant if the FDR < 0.05 and their EICs indicated reasonable quality. For pathway enrichment, we again restricted our examination to pathways with 3 matching nodes and enrichment scores, s, < 0.10. Our stringent standards likely also hide greater breadth of true human metabolic response to TRP exposure. Relaxing the FDR criterion to < 0.20 would not have appreciably changed the results of our Exposure MWAS findings. However, relaxing the FDR of ΔBiomarkers models would have greatly expanded the number of potential pathways to consider at the expense of specificity to traffic exposure.

Only three variables representing particulate metal exposures were associated with metabolic perturbations. Other widely used TRP parameters, such as PM_2.5_ mass and PNC, had no association. We explored categorical exposures (e.g., Highway vs. Non-commute exposure scenario) (*data not shown*) and found no significant associations up to an FDR of < 0.20. In our view, rich exposure characterization, which included metal content, at the microenvironmental level aided in our discovery, but also spoke to the importance of actual pollutant measurement in similar human observation designs. Perturbations in plasma metabolomes as a result of ambient air pollution exposures have recently been reported using targeted [51] and untargeted [31] metabolomic profiling. The latter, like the current study, used repeated biological measures and detailed exposure characterization, including trace metal composition, to capture the dynamic nature of high-dimensional exposure and biological responses.

Finally, it is worth reiterating the interpretive challenges involved in an untargeted, HRM analysis. Extracted features are typically matched by m/z alone to annotation and network databases. This results in ‘many-to-many’ matches of features to compounds in the databases. For example, 2,716 features, less than 20% of the total supplied to mummichog (n = 14,282) for Pb-based enrichment, matched 7,309 compounds on the human metabolic network. This expansion is then reduced by network analysis, vastly improving interpretability [29]. Nevertheless, there remains a large proportion of unknown metabolites that are changing in response to Pb exposure. Relatedly, the use of m/z alone for matching also belies degeneracy—the presence of isotopic versions of features. Two features may be isotopes of the same metabolite but may match to separate compounds in high-resolution online databases. However, in our approach, we filtered significant features from the MWAS through examination of extracted ion chromatograms. Annotation and pathway analysis was performed on features that had clear, dominant peaks with reasonable tolerance of isotopic peaks. We believe this served to reduce the chance of misspecification of feature annotation. Like others at the stage of pathway enrichment, our data provide strong suggestions of pathway level perturbations, but feature validation is ultimately necessary. Before the substantial investment in chemical standards and reanalysis of samples, examining correlations between leading features and hypothesized, observed outcomes may provide confidence in the putative identification of important compounds [52]. We explored the correlations of the putative 20-OH-LTB4 features with the remainder of the measured metabolome and clinical changes in neutrophils or eosinophils from complete blood counts in our population and found no significant associations (*results not shown*). We found either no further support for leukotriene metabolite identifications, or, perhaps, observed changes that were subclinical and focused. Either definitive identification of specific features using MS/MS or reliable reference standards can improve the certainty of our results.

Collectively, we believe the results of this analysis provide evidence that HRM can serve as a sensitive tool of external, air pollution exposures, when used within a quasi-experimental human panel. Our environmental measures went beyond standard air pollution sampling practices used in large population studies. These advantages in study design and data capture enhanced our ability to capture changes in human metabolism with respect to TRP exposure. While there is additional support in the air pollution literature of acute oxidative stress response in humans mediated via exposures to particulate metal exposures, detection of this response using metabolomic profiling around traffic related exposures warrants replication. The general inconsistencies of inflammatory markers and their associations with certain PM_2.5_ components necessitate additional studies to provide added confidence in our current findings. For the purposes of bioeffect screening, plasma HRM was useful in providing interpretable results. Further study should improve upon repeated sampling of plasma with shorter windows and multi-day sampling after single exposures in a human population. If our metabolomic sampling of plasma echoed the sampling of dried blood spots—used for targeted biomarkers in this study—then perhaps pro- and anti-inflammatory processes associated with TRP can be tracked over time. Finally, while much of air pollution toxicology research focuses on proinflammatory markers of response, we show cause to study pro-resolving mediators as well. For example, targeting eicosanoids, specifically LTB4, in a future iteration of a traffic exposure metabolomics study may improve our understanding of the interplay of systemic inflammation in acute response.

## Conclusions

HRM can detect plausible changes in the plasma metabolome in response to traffic related pollution in a commuter panel study. In-vehicle particulate metal exposures were associated with within-day perturbations of several pathways; however, one metabolic pathway, leukotriene metabolism, was also associated to changes in proinflammatory cytokines.

## Supporting information

S1 Table Targeted biomarker levels at pre-commute measurement, by commute type. (DOCX)

S2 Table Selected metabolite measures at pre-commute samplings. (DOCX)

S1 Fig Venn diagram of features in negative mode ionization associated with variability in in-vehicle Al, Fe, and Pb exposures. (PNG)

S2 Fig Venn diagram of features in negative mode ionization associated with pre- to post-exposure changes in inflammatory markers. (PNG)

S3 Fig Manhattan plots of associations between changes in negative ionization mode feature intensities with changes in targeted biomarkers (ΔBiomarkers). (TIF)

S4 Fig Manhattan plots of associations between changes in positive ionization mode feature intensities with in-vehicle, traffic-related pollutants. (TIF)

S5 Fig Manhattan plots of associations between changes in positive ionization mode feature intensities with changes in targeted biomarkers (ΔBiomarkers). (TIF)

S1 Datasets Package file with exposure concentrations, positive ionization mode feature table, negative ionization mode feature table, and accompanying data dictionary. (ZIP)
