# Supplementary material for: Particulate metal exposures induce plasma metabolome changes in a commuter panel study
Source: PLoS One. 2018 Sep 19;13(9):e0203468. doi: 10.1371/journal.pone.0203468 (PMC6145583; doi:10.1371/journal.pone.0203468)
Supplement: S1 Table — (DOCX) [file pone.0203468.s001.docx]

| **S1 Table: Targeted biomarker levels at pre-commute measurement, by commute type** | | | | | | | | | |
| --- | --- | --- | --- | --- | --- | --- | --- | --- | --- |
|  | hs-CRP | TNF-α | IL1β | IL6 | IL8 | sICAM | sVCAM | eNO (ppb) | FEV1  (% pred.) |
| *Highway* |  |  |  |  |  |  |  |  |  |
| N | 35 | 35 | 35 | 21 | 35 | 35 | 35 | 35 | 35 |
| Mean (SD) | 12,208 (32,917) | 0.09 (0.07) | 1.94 (4.62) | 0.07 (0.21) | 5.46 (5.44) | 1,455 (1,459) | 3,129 (2,622) | 25.37 (20.25) | 89.86 (12.69) |
| Median (IQR) | 2,549 (5,294) | 0.08 (0.04) | 0.84 (1.66) | 0.04 (0.05) | 4.58 (1.56) | 895  (823) | 2,116 (2,459) | 21  (15) | 91  (14) |
|  |  |  |  |  |  |  |  |  |  |
| *Non-Highway* |  |  |  |  |  |  |  |  |  |
| N | 34 | 34 | 34 | 16 | 34 | 34 | 34 | 33 | 34 |
| Mean (SD) | 11,270 (16,267) | 0.08 (0.06) | 1.51 (2.13) | 0.04 (0.08) | 4.51  (1.80) | 1,487 (1,453) | 3,362 (2,787) | 22.18 (19.95) | 88.21 (11.94) |
| Median (IQR) | 3,263 (10,917) | 0.07 (0.04) | 0.96 (1.33) | 0.04 (0.05) | 4.33 (1.81) | 821 (1,540) | 2,475 (2,509) | 17  (16) | 92  (17) |
| ^a^ Indicates p-value significant at α < 0.05 through Student’s t-test for comparing across commute types;  Units are pg/mL unless noted otherwise;  ppb signifies parts per billion;  % pred. is percent of predicted value | | | | | | | | | |
