# Supplementary material for: Particulate metal exposures induce plasma metabolome changes in a commuter panel study
Source: PLoS One. 2018 Sep 19;13(9):e0203468. doi: 10.1371/journal.pone.0203468 (PMC6145583; doi:10.1371/journal.pone.0203468)
Supplement: S2 Table — (DOCX) [file pone.0203468.s002.docx]

| **S2 Table: Selected metabolite measures at pre-commute samplings** | | | | | | |
| --- | --- | --- | --- | --- | --- | --- |
|  | **Non-Asthmatics** | | | **Asthmatics** | | |
| *Metabolite* | *N* | *Mean* | *SD* | *N* | *Mean* | *SD* |
| Amino Acid |  |  |  |  |  |  |
| Alanine ^a^ | 34 | 446.49 | (7.19) | 39 | 441.97 | (7.58) |
| Arginine ^a^ | 34 | 87.31 | (1.67) | 39 | 85.44 | (1.91) |
| Asparagine ^a^ | 34 | 7.13 | (0.11) | 39 | 7.06 | (0.12) |
| Citrulline | 34 | 10.41 | (0.17) | 39 | 10.32 | (0.22) |
| Glutamate | 34 | 157.91 | (4.80) | 39 | 155.89 | (7.26) |
| Glutamine | 34 | 61.70 | (0.59) | 39 | 61.36 | (0.73) |
| Histidine ^a^ | 34 | 124.82 | (1.37) | 39 | 123.72 | (1.22) |
| Isoleucine/Leucine | 34 | 122.44 | (1.62) | 39 | 121.53 | (1.82) |
| Lysine ^a^ | 34 | 348.41 | (4.60) | 39 | 344.13 | (5.41) |
| Methionine ^a^ | 34 | 29.79 | (0.44) | 39 | 29.50 | (0.47) |
| Phenylalanine ^a^ | 34 | 79.83 | (1.57) | 39 | 78.24 | (2.85) |
| Proline | 34 | 278.95 | (5.95) | 39 | 275.61 | (7.67) |
| Serine | 34 | 93.03 | (1.41) | 39 | 92.64 | (1.27) |
| Threonine | 34 | 123.40 | (2.12) | 39 | 122.89 | (2.27) |
| Tryptophan | 34 | 33.62 | (0.42) | 39 | 33.34 | (0.58) |
| Tyrosine ^a^ | 34 | 61.80 | (0.94) | 39 | 61.12 | (1.07) |
| Amino Acid Metabolites |  |  |  |  |  |  |
| Kynurenine | 34 | 2.01 | (0.03) | 39 | 2.00 | (0.04) |
| Oxoproline | 34 | 35.48 | (0.47) | 39 | 35.19 | (0.80) |
| Exogenous Chemical |  |  |  |  |  |  |
| Caffeine | 34 | 74.26 | (3.49) | 39 | 73.45 | (3.54) |
| FA Metabolism |  |  |  |  |  |  |
| a-Linolenic acid | 34 | 106.98 | (2.21) | 39 | 107.11 | (2.75) |
| Acetyl-carnitine | 34 | 1.78 | (0.08) | 36 | 1.77 | (0.11) |
| Carnitine ^a^ | 34 | 27.98 | (0.70) | 39 | 27.12 | (1.35) |
| Linoleic acid | 34 | 1137.79 | (12.23) | 39 | 1138.04 | (14.74) |
| Health Indicators |  |  |  |  |  |  |
| Cholesterol | 3 | 1546.00 | (104.16) | 2 | 1432.60 | (41.03) |
| Creatine | 33 | 37.06 | (1.89) | 38 | 36.52 | (1.20) |
| Creatinine | 34 | 72.86 | (0.95) | 39 | 72.98 | (0.77) |
| Lipid Metabolism |  |  |  |  |  |  |
| Choline | 34 | 1.26 | (0.01) | 39 | 1.27 | (0.02) |
| Sphinganine | 13 | 0.03 | (0.00) | 20 | 0.03 | (0.00) |
| Values are Mean (SD) in µM;  Sample sizes (N) represent samplings, not individual participants;  ^a^ denotes statistical significance with FDR < 0.05 between Asthmatics and Non-Asthmatics; FA = Fatty Acid | | | | | | |
