# Supplementary figures and images for: Particulate metal exposures induce plasma metabolome changes in a commuter panel study
Source: PLoS One. 2018 Sep 19;13(9):e0203468. doi: 10.1371/journal.pone.0203468 (PMC6145583; doi:10.1371/journal.pone.0203468)

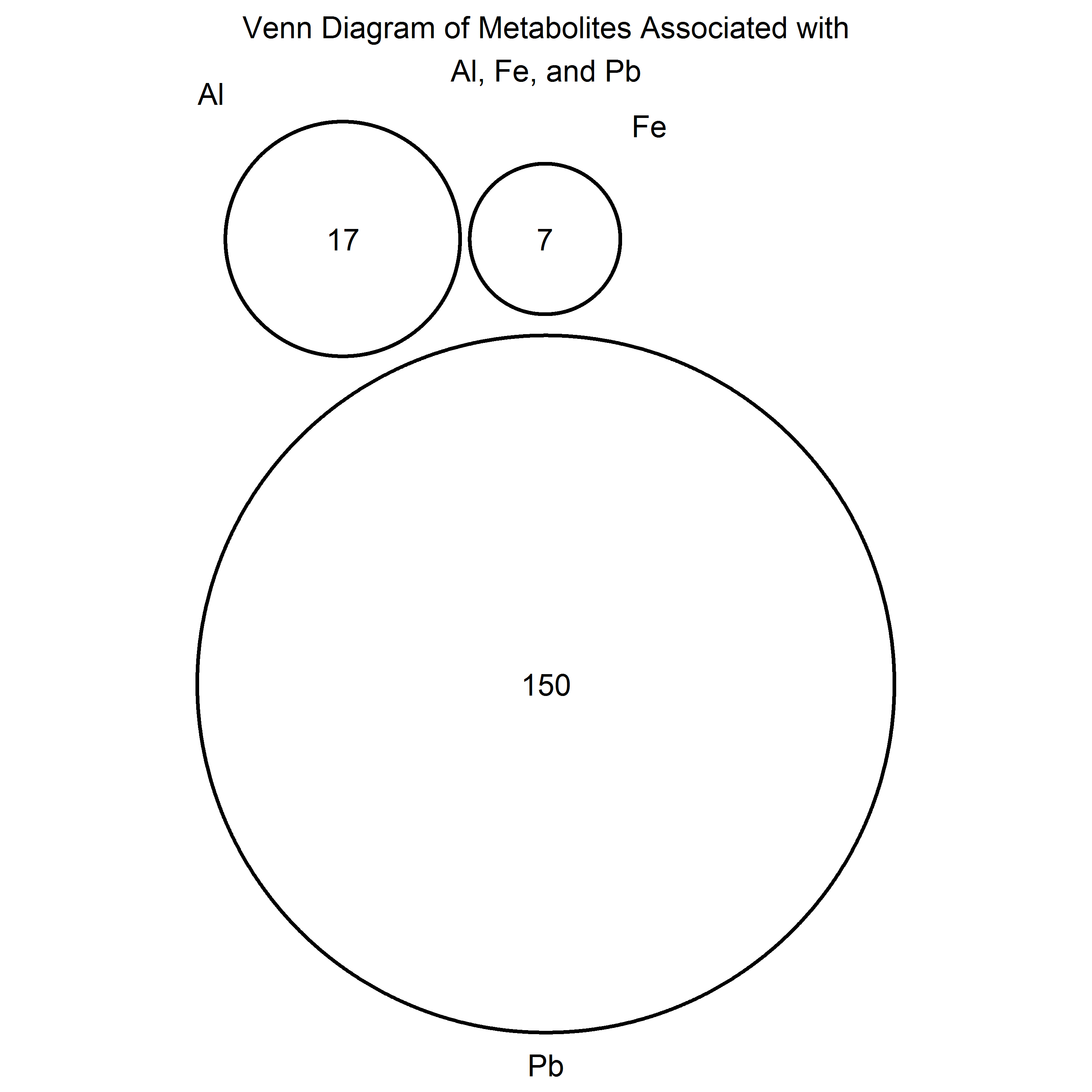

Supplement: S1 Fig — (PNG) [file pone.0203468.s003.png]

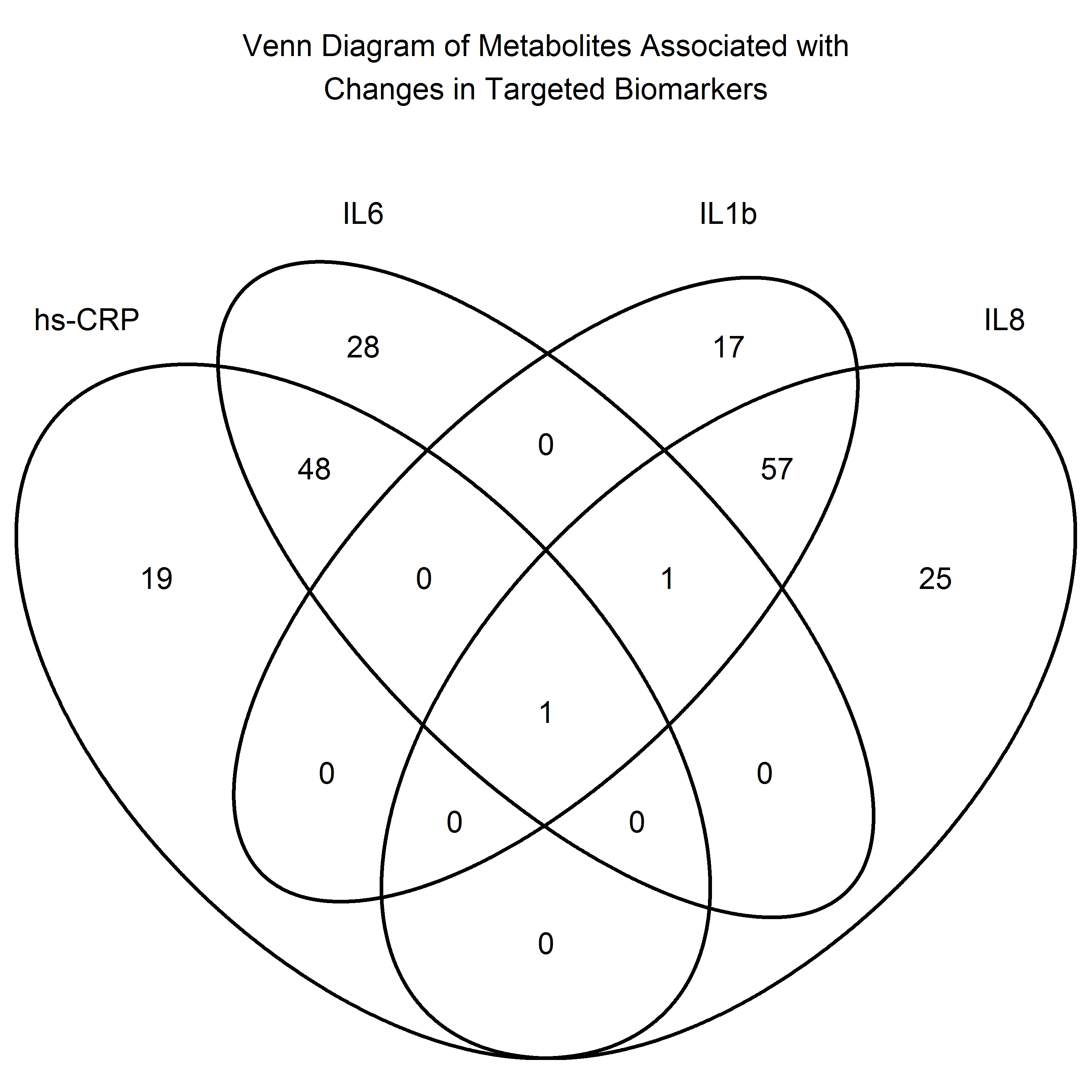

Supplement: S2 Fig — (PNG) [file pone.0203468.s004.png]

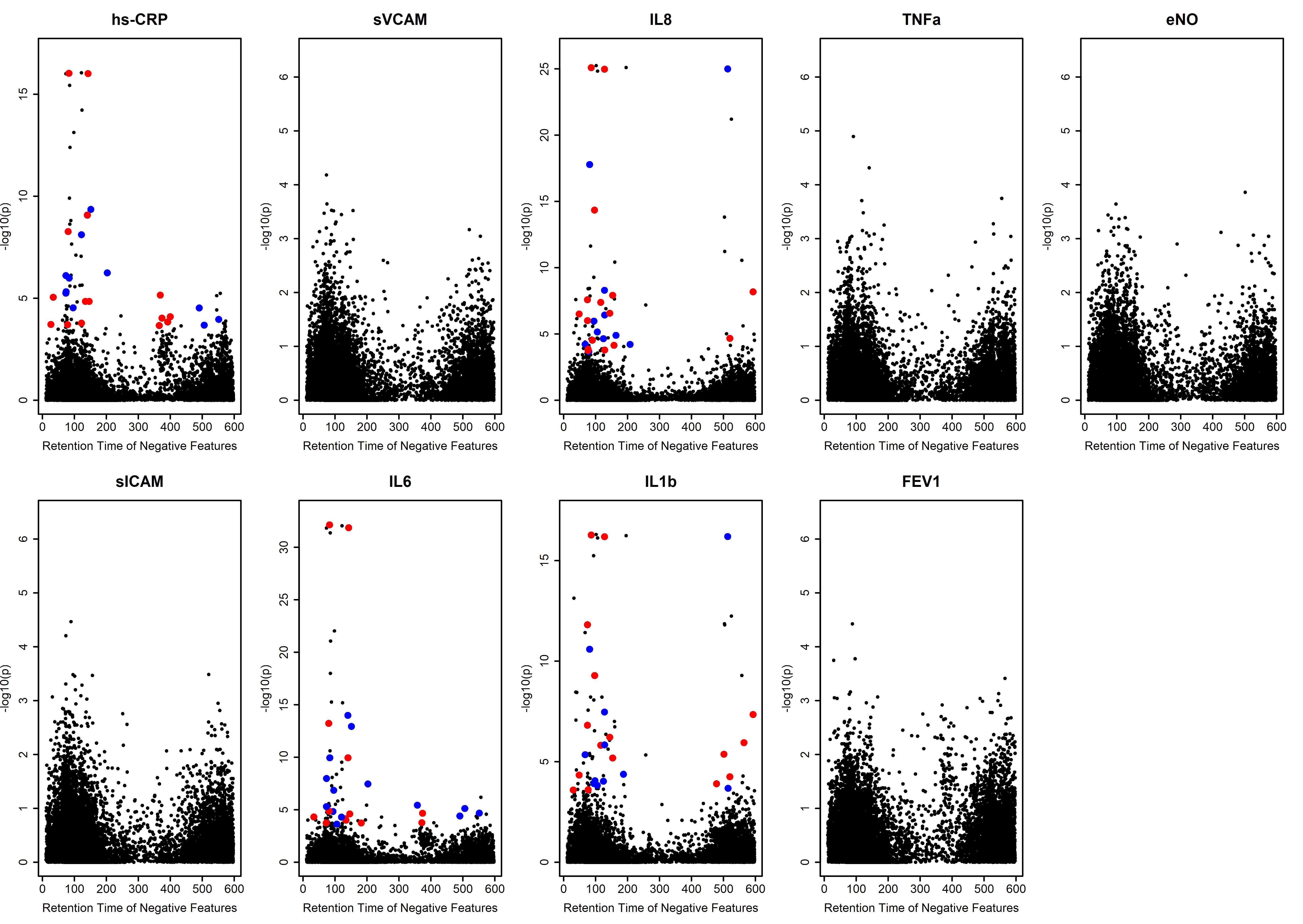

Supplement: S3 Fig — (TIF) [file pone.0203468.s005.tif]

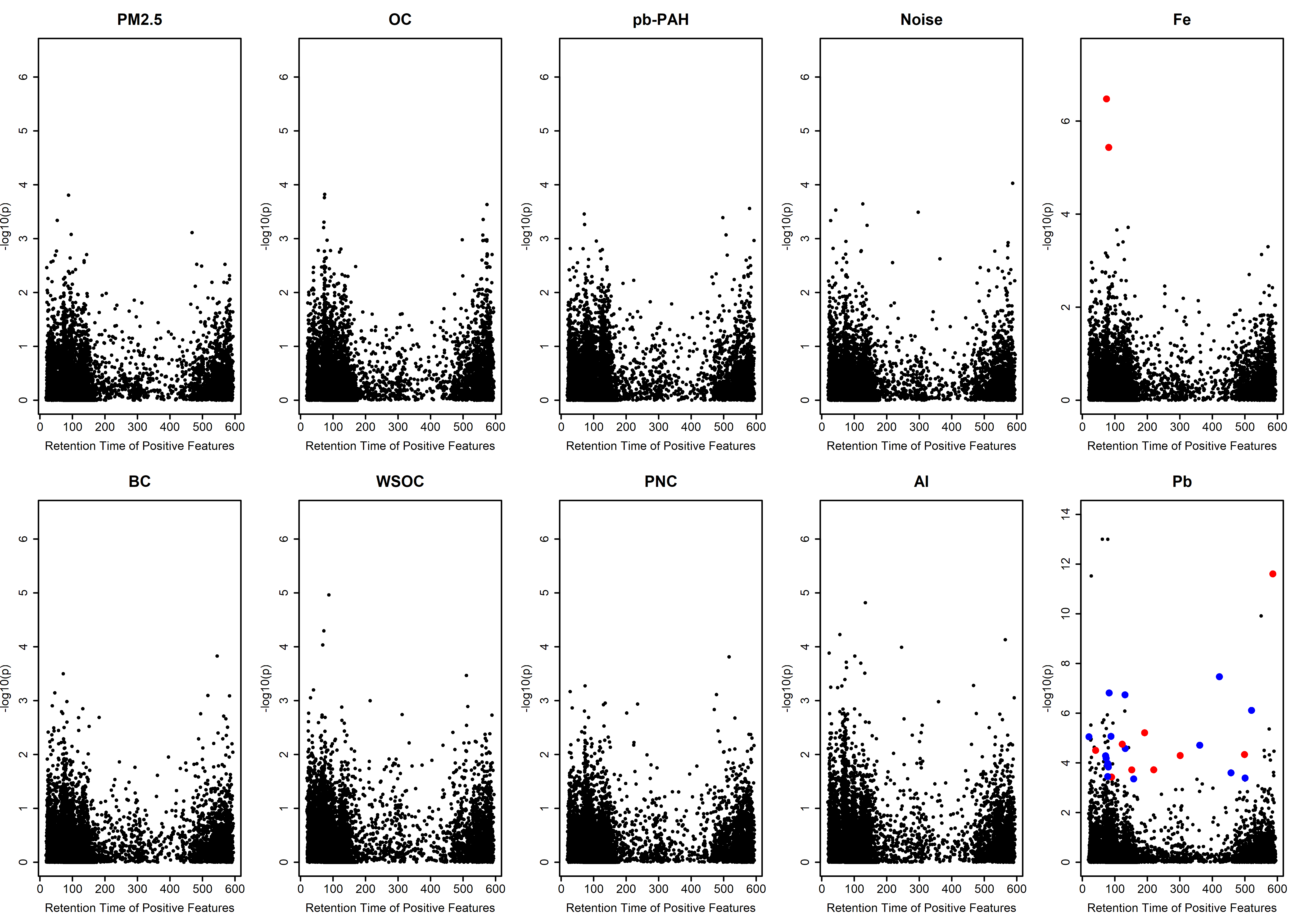

Supplement: S4 Fig — (TIF) [file pone.0203468.s006.tif]

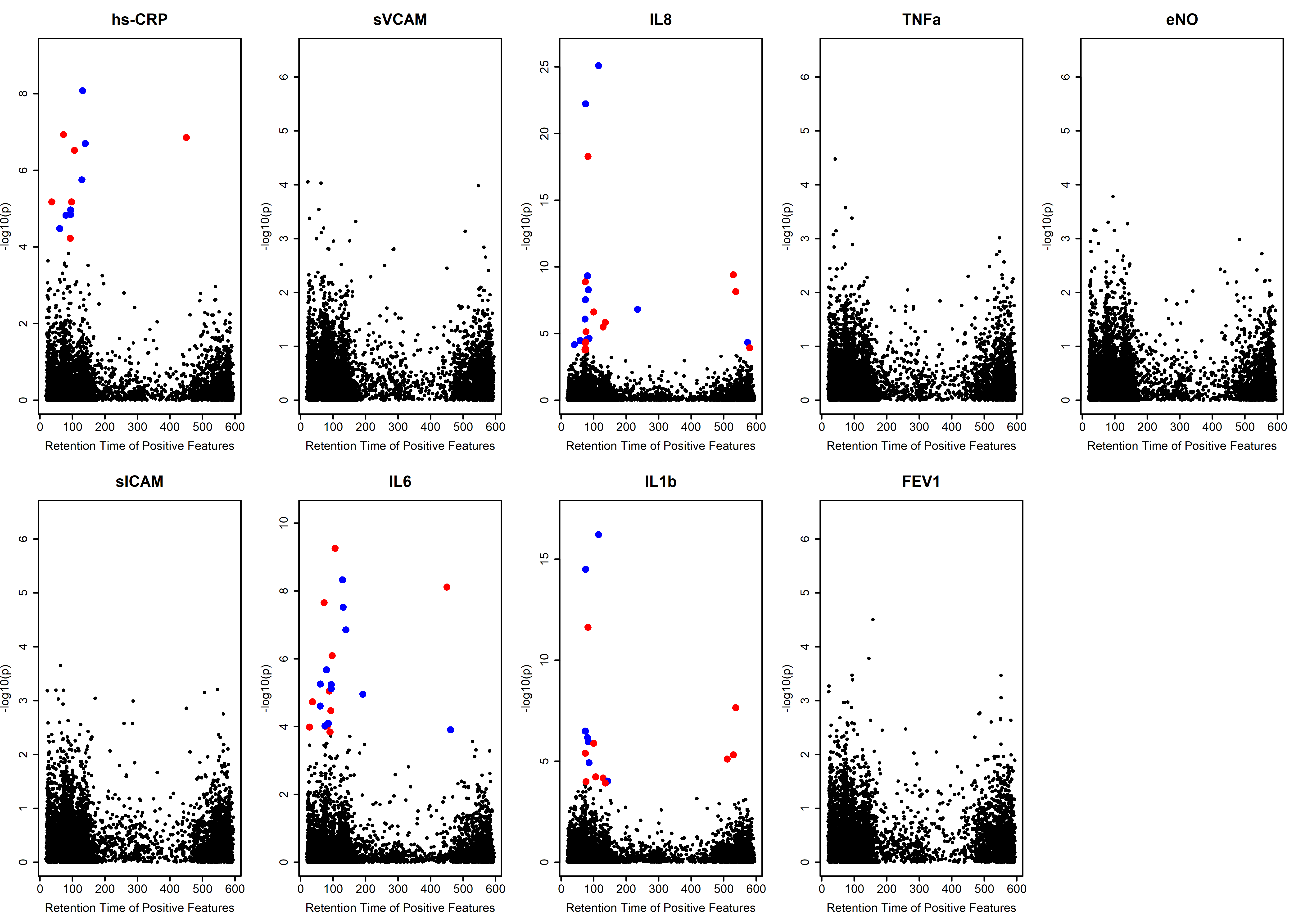

Supplement: S5 Fig — (TIF) [file pone.0203468.s007.tif]
